# Development of a Microsoft Excel tool for applying a factor retention criterion of a dimension coefficient to a survey on patient safety culture

**DOI:** 10.1186/s12955-017-0784-8

**Published:** 2017-10-27

**Authors:** Tsair-Wei Chien, Yang Shao, Dong-Hui Jen

**Affiliations:** 1 Department of Medical Research, Chi-Mei Medical Center, Tainan, Taiwan; 2 Department of Hospital and Health Care Administration, Chia-Nan University of Pharmacy and Science, Tainan, Taiwan; 3 Department of Electronics and Information Engineering, Tongji Zhejiang College, Jiaxing, China; 4 Department of Chinese Medicine, Chi-Mei Medical Center, No. 901, Chung Hwa Road, Yung Kung Dist, Tainan, 710 Taiwan

**Keywords:** Dimension coefficient, Patient safety culture survey, Visual basic for applications, Area under receiver operating characteristic curve, Parallel analysis

## Abstract

**Background:**

Many quality-of-life studies have been conducted in healthcare settings, but few have used Microsoft Excel to incorporate Cronbach’s α with dimension coefficient (DC) for describing a scale’s characteristics. To present a computer module that can report a scale’s validity, we manipulated datasets to verify a DC that can be used as a factor retention criterion for demonstrating its usefulness in a patient safety culture survey (PSC).

**Methods:**

Microsoft Excel Visual Basic for Applications was used to design a computer module for simulating 2000 datasets fitting the Rasch rating scale model. The datasets consisted of (i) five dual correlation coefficients (correl. = 0.3, 0.5, 0.7, 0.9, and 1.0) on two latent traits (i.e., true scores) following a normal distribution and responses to their respective 1/3 and 2/3 items in length; (ii) 20 scenarios of item lengths from 5 to 100; and (iii) 20 sample sizes from 50 to 1000. Each item containing 5-point polytomous responses was uniformly distributed in difficulty across a ± 2 logit range. Three methods (i.e., dimension interrelation ≥0.7, Horn’s parallel analysis (PA) 95% confidence interval, and individual random eigenvalues) were used for determining one factor to retain. DC refers to the binary classification (1 as one factor and 0 as many factors) used for examining accuracy with the indicators sensitivity, specificity, and area under receiver operating characteristic curve (AUC). The scale’s reliability and DC were simultaneously calculated for each simulative dataset. PSC real data were demonstrated with DC to interpret reports of the unit-based construct validity using the author-made MS Excel module.

**Results:**

The DC method presented accurate sensitivity (=0.96), specificity (=0.92) with a DC criterion (≥0.70), and AUC (=0.98) that were higher than those of the two PA methods. PA combined with DC yielded good sensitivity (=0.96), specificity (=1.0) with a DC criterion (≥0.70), and AUC (=0.99).

**Conclusions:**

Advances in computer technology may enable healthcare users familiar with MS Excel to apply DC as a factor retention criterion for determining a scale’s unidimensionality and evaluating a scale’s quality.

**Electronic supplementary material:**

The online version of this article (10.1186/s12955-017-0784-8) contains supplementary material, which is available to authorized users.

## Background

In healthcare, the degree of patient harm was first publicized in the 1990s [1]. After the book *To err is human: building a safer health system* [2] was released, 474 papers were published on Medline using the keyword of patient safety culture (PSC) to search as of June 3, 2017. Safety experts [3–5] addressed that patient safety begins with the enforcement of system safety of healthcare organizations, and this culture is a fundamental factor that influences healthcare system safety [4].

Many studies [6–8] have used the Safety Attitudes Questionnaire [9] as a tool to verify PSC reliability and validity [6, 10]. However, the comparison in practice is commonly made between departments in a hospital. Few studies examine the reliability and validity of PSC on a department-unit base. Person misfit indicator is commonly used in the literature [11] to identify person possible carefulness and careless behavior in response. If data are not purified or polished, the comparison or analysis is meaningless because a quality scale must be one-dimensional, or all variables loading on the same factor should make sense when scores are summed. The first research question is how to easily examine unit-based construct validity.

**Fig. 1:**
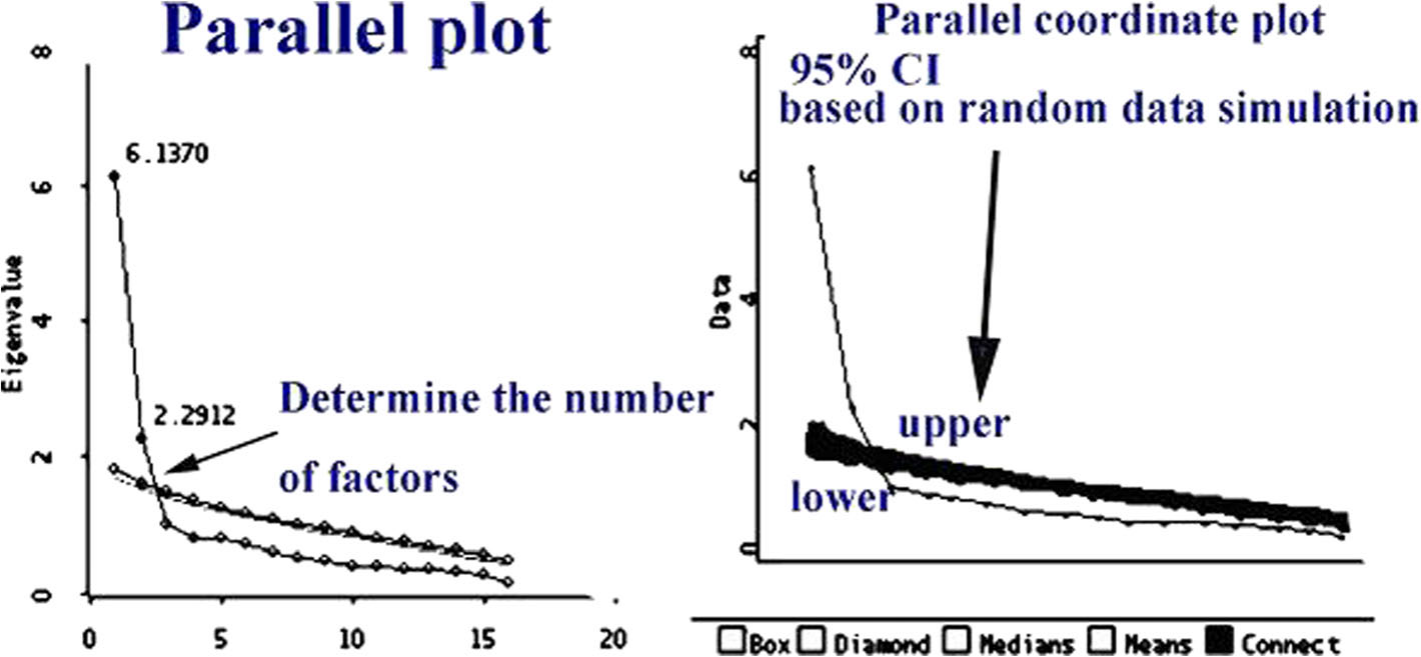
View of the scree parallel plot and scree simulation for determining the number of factors to retain

Psychological characteristics are defined as an abstract or latent nature rather than a tangible and observable entity [12–14]. A summation score across items on a domain is meaningless if items measure different features. The most top priority for an analysis is “determining the number of factors to retain”, although certain considerations are made in exploratory factor analysis (EFA) [15–17] including Kaiser’s rule (factors with eigenvalue >1), Scree plot criteria, and variance explained criteria. Horn’s Parallel analysis (PA) [18] has been reported to be the best method [19–21]. The number of factors is determined where the eigenvalue in the random data is lower than the respective component of the actual data (Fig. 1) [22, 23]. However, PA is not implemented in commonly used statistical software (e.g., SPSS and SAS). A user-friendly website [24, 25] and the author-made macro applied to SAS [26] have been recommended. It is unheard for use on Microsoft Excel.

A dimension coefficient (DC) has also been proposed in a previous study [27] using Rasch model [28]. We are interested in further comparing the two aforementioned methods of PA and DC that can be combined for use in practice.

The present study aimed to identify (1) why Cronbach’s α (i.e., internal consistency reliability) is not a sufficient condition of validity [29, 30]; (2) whether DC can be incorporated with PA for precisely inspecting a one-dimensional scale; and (3) how a module in Microsoft Excel can be used for examining the validity of a PSC domain on a unit base by checking the DC.

## Methods

### Simulative datasets

Using Microsoft Excel Visual Basic for Applications, we designed a computer module to manipulate 2000 datasets fitting the Rasch rating scale model (i.e., like 5-point Likert-type scale) [31], which consisted of (i) five correlation coefficients (correl. = 0.3, 0.5, 0.7, 0.9, and 1.0) on two latent traits (i.e., true scores) following a normal distribution and answering their respective 1/3 and 2/3 items; (ii) 20 scenarios of item lengths from 5 to 100; and (iii) 20 sample sizes from 50 to 1000. Each item containing 5-point polytomous responses (similar to the PSC format) was uniformly distributed in difficulty across a ± 2 logit range. Three methods (i.e., dimension interrelation ≥0.7, Horn’s PA 95% CI, and individual random eigenvalues) were used to determine one factor to retain.

### Simulation process

When person true scores and item difficulties are known, we can simulate Rasch data [32]. Scale’s reliability and DC were simultaneously calculated for each simulative dataset, where reliability is defined as Cronbach’s α. DC is expressed in a previous study [27, 33, 34].

The upper 95% CIs of an eigenvalue in the random simulative dataset can be used for determining the number of factors [23] using the website link [25] or Vista software [35]. In this study, the eigenvalues of PA 95% CI were extracted from the website [25] (see Additional file 1 [extracting data from website] and Additional file 2 [simulation process]). Box plots were applied to show their dispersions of Cronbach’s α and DC (on y-axis) across five scenarios (on x-axis) of dimension correlation (correl. = 0.3, 0.5, 0.7, 0.9, and 1.0). The first research question (i.e., Cronbach’s α is not sufficient to a scale’s validity) could be verified by the plot comparison.

In literature, Tennant and Pallant [36] addressed that the Rasch fit statistics performed poorly (i.e., identify two domains) where dimensions were interlaced with equal item length and where the correlation between factors was near 0.7. If two dimensions (corr. ≈ 0.7) are interlaced with only 1/3 items, we can reasonably consider the scale as one dimension.

The accuracy of the three methods (i.e., dimension interrelation ≥0.7, PA 95% CIs, and individual random eigenvalues) was compared to determine the number of factors (i.e., 1 denotes one factor and 0 represents many factors maked with dots in Additional file 3). Indicators included sensitivity, specificity, and area under the receiver operating characteristic curve (AUC). The second question (i.e., DC can be combined with PA for inspecting the number of factors for a scale) can then be answered.

### Demonstrations of actual PSC data

**Fig. 2:**
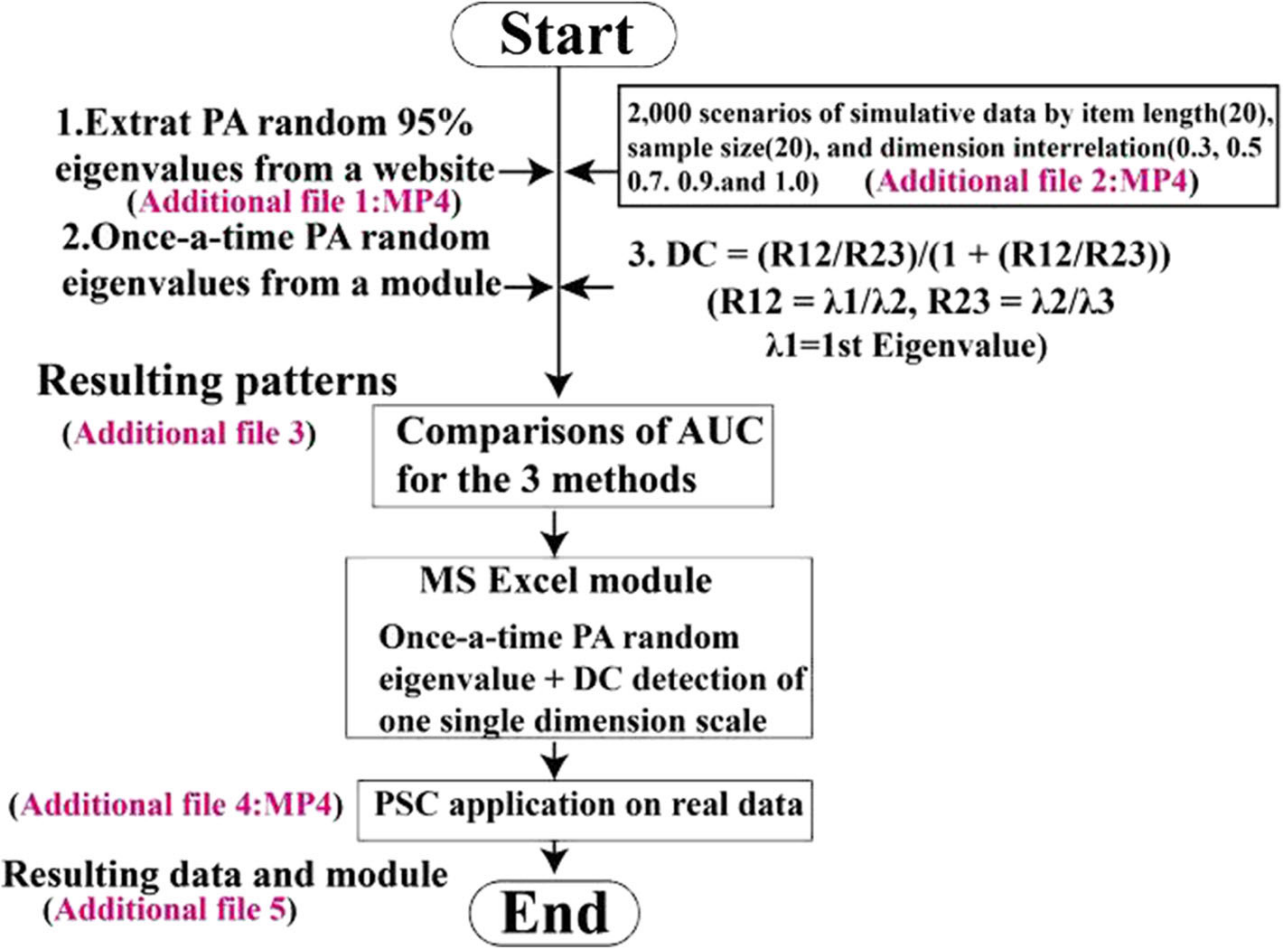
Study flowchart

To answer the third research question, we collected data from previous studies regarding a PSC survey in a hospital [6, 7, 37]. The sample size was 2237 with 97 departments. The PSC questionnaire comprised six domains with 30 items. The domains were teamwork climate (D1, 6 items), safety climate (D2, 7 items), job satisfaction (D3, 5 items), recognition of depression (D4, 4 items), perception of management (D5, items), and working conditions (D6, items). An author-made MS Excel module was applied to examine the validity of PSC in a unit base. All computations for the unit DC and Cronbach’s α were subjected to a sample size > = 10. The study flowchart is shown in Fig. 2.

### Statistical analysis

SPSS 18.0 for Windows (SPSS Inc., Chicago, IL, USA) and MedCalc 9.5.0.0 for Windows (MedCalc Software, Mariakerke, Belgium) were used to draw box plots and AUC curves. In the author-made MS Excel module, two methods of DC and PA were used to determine the number of factors. In addition, a scree plot was drawn.

## Results

### Task 1: Cronbach’s α is insufficient to determine a scale’s validity

**Fig. 3:**
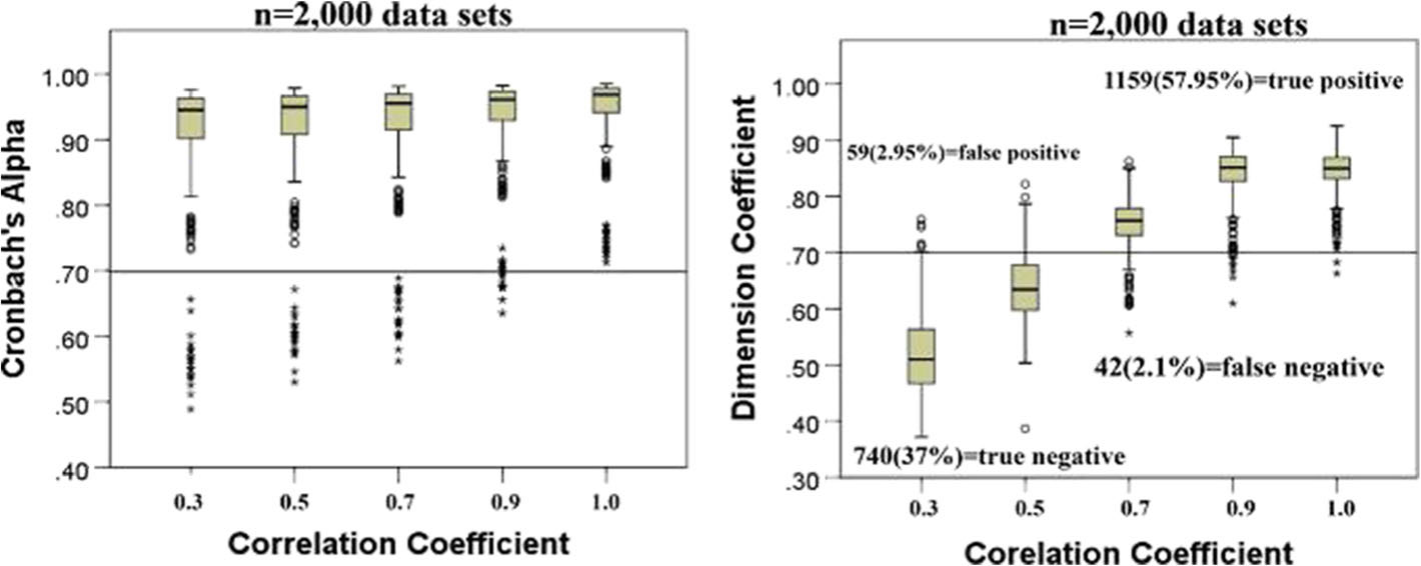
Cronbach’s alpha and DC related to the dimension interrelation

Figure 3 (left) shows that most values of Cronbach’s α were greater than 0.80 regardless of the degree of dimension interrelation (on x-axis). The long item length increased the value of Cronbach’s α according to the Spearman–Brown prediction formula [38]. The criterion at 0.70 (on y-axis), which represented an acceptable quality of scale, was improbable. Some data with 1/3 proportion of items with a low dimension interrelation (=0.3) yielded a high Cronbach’s α, as shown in the first bin of Fig. 3 (left).

In Fig. 3 (right), 59 (2.95%) cases were located at the left top (false positive) part, 42 (2.1%) were at the right bottom (false negative) part, 1159 (57.95%) were at the right top (true positive) part, and 740 (37%) were found at the left bottom (true negative) part. This pattern shows that a high degree of dimension interrelation indicated strong DC tendency. A cutting point of 0.7 (on y-axis) suggested that DC could exactly separate two domains (i.e., top and bottom) based on our simulation data.

### Task 2: DC combined with PA to inspect the number of factors for a scale

**Fig. 4:**
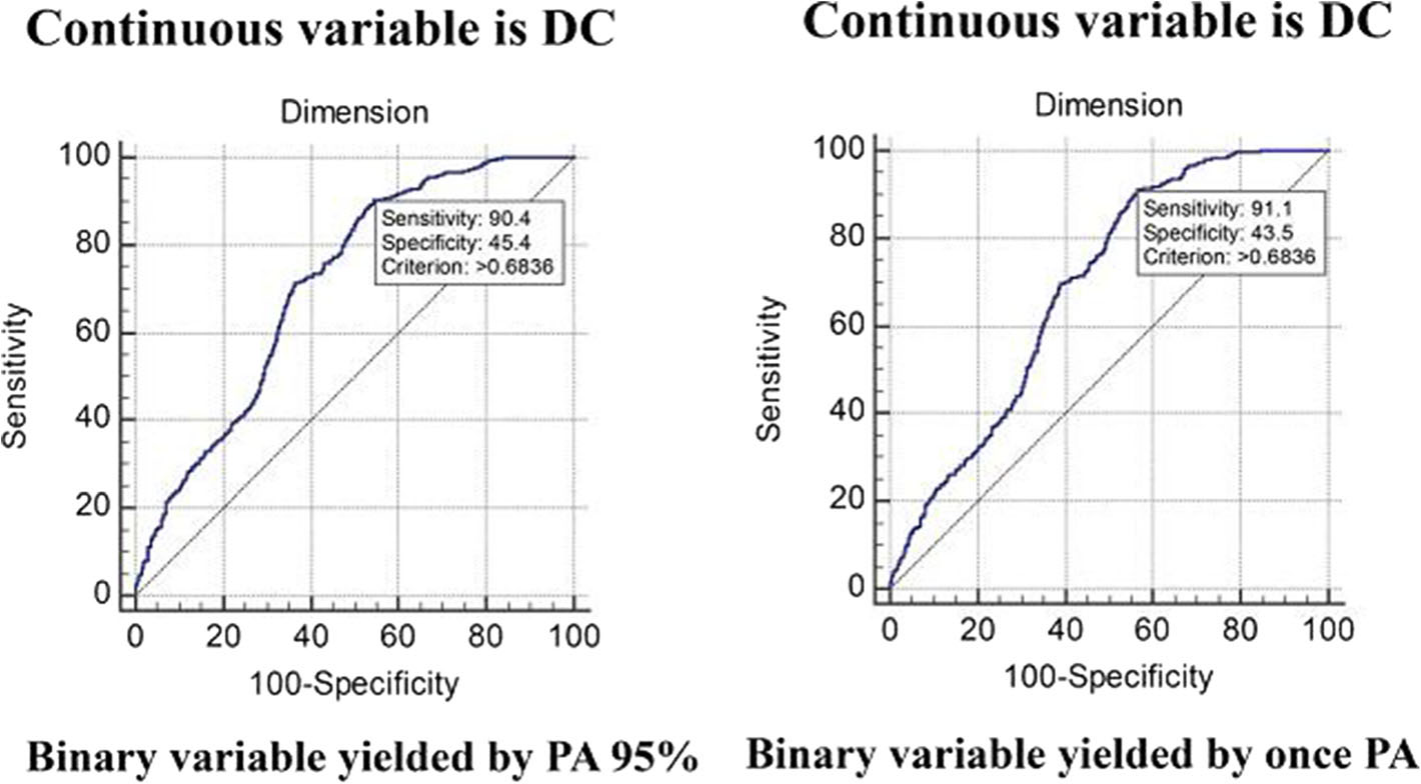
Comparison of AUCs of 0.71 (left) and 0.68 (right) by two PA methods

Continous variable DCs (y-axis) were combined with a binary variable classified by checking PA 95 CIs and individual random eigenvales along with dimension interrelations (x-axis) (Fig. 4).

**Fig. 5:**
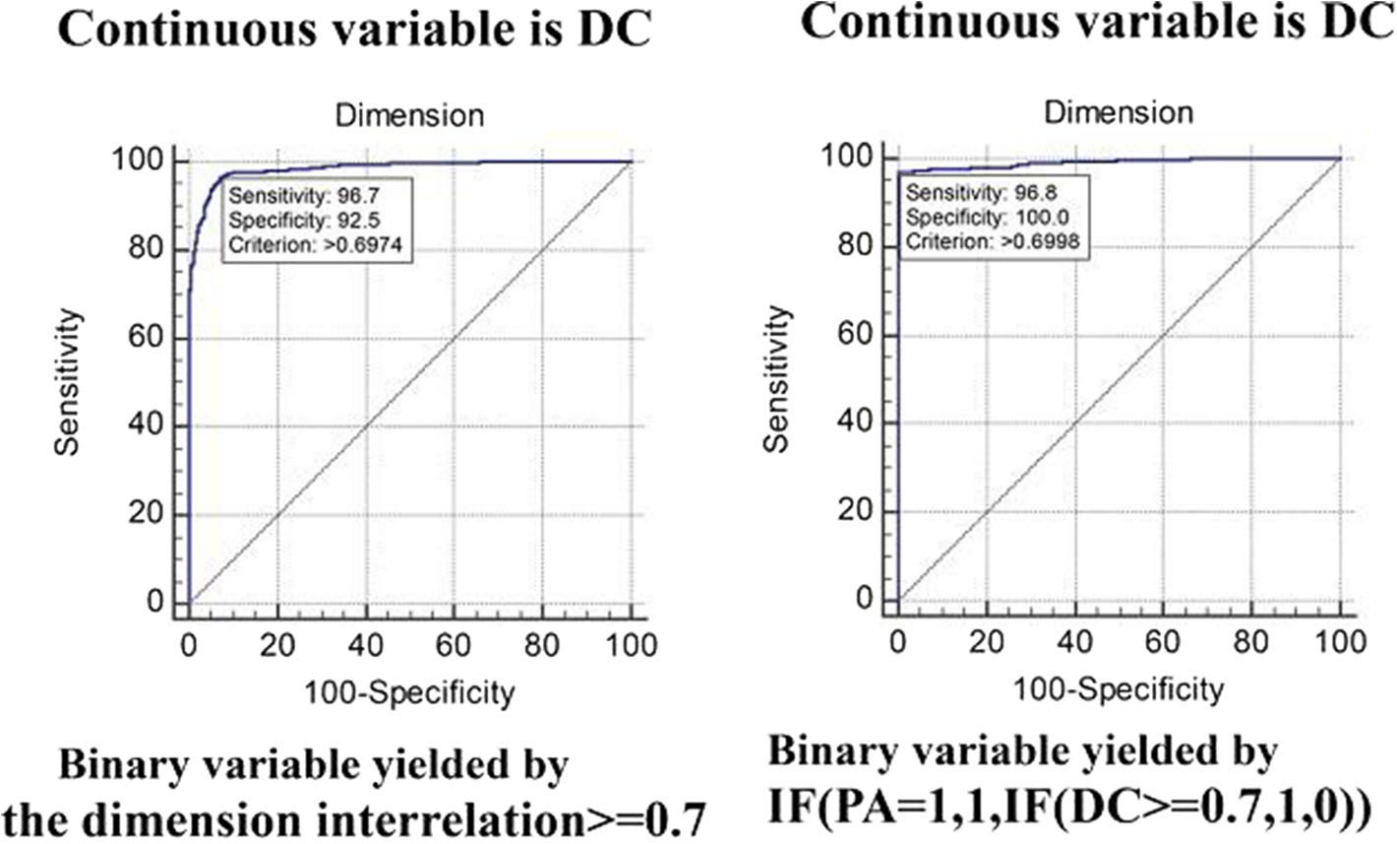
DC (AUC = 0.98) combined with PA (AUC = 0.71) for inspecting the number of factors for a scale with AUC = 0.99

The two methods demonstrated equivalent sensitivity (90%:91%), specificity 45%:43%), and AUC (0.71:0.68) with a common DC criterion at 0.68 (Fig. 4), which was not competitive to the counterpart with known information (i.e., dimension interrelation ≥0.7 was defined one-dimensional) in Fig. 5 (left). Thus, a criterion for DC at 0.70 presented 96% sensitivity, 92% specificity, and AUC = 0.98.

After combining PA and DC with a tranformation rule [i.e., by a MS Excel equation = IF (PA determination = 1, 1, IF (DC ≥ 0.7, 1, 0), where 1 denotes one factor and 0 represents many factors maked with dots in Additional file 3), the specificity and AUC could be improved (up to 100 and 0.99, respectively), but the sensitivity was unchanged. The DC criterion was still located at 0.70 (Fig. 5, right).

A comparison of the results in Additional file 3 revealed that the two PA methods led to misclassification on two situations: (i) short item length (=5 items) and small sample size (=50) yielded false positive (i.e., two factors are classified as one factor); and (ii) long item length (>40 items) and large sample size (>200) were prone to false negative (i.e., one factor is grouped into many factors).

### Task 3: MS excel module is an easy way to examine the validity of PSC

All DCs and Cronbach’s α in hospital units could be easily computed within 30 s for a domain (see Additional file 4). Table 1 shows that some DCs and Cronbach’s α were less than 0.7, thereby indicating that data entries should be purified or polished prior to analysis. Such data may be due to cheating, careless behaviors, or other reasons in responses. The global subscales with low construct validity (<0.70, see the first row in Table 1) were teamwork climate (0.58) and working conditions (0.66), indicating that some misfit items existed in the datasets. Whether a differential item functioning (DIF) [39] phenomenon is emerging among hospitals needs further clarification is required to futhrer investigate, The term of DIF shows the extent to which a specific item might be measuring different features for members of separate subgroups, such as members from different types of hospital in this study.

**Table 1:**
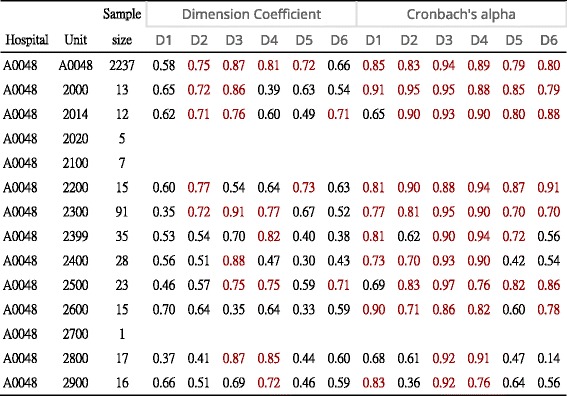
Unit-based DC and Cronbach’s alpha computed by the author-made module

Note. All computations are subject to the sample size > = 10; The domains include teamwork climate (D1, 6 items), safety climate (D2, 7 items), job satisfaction (D3, 5 items), recognition of depression (D4, 4 items), perception of management (D5, items), and working conditions (D6, items)

## Discussion

### Principal findings

Our most important finding was that (1) Cronbach’s α was not a sufficient condition of validity; (2) DC is an essential complement to PA for inspecting whether a scale is one single construct; and (3) a module in Microsoft Excel is presented as an easy approach for examining the validity of PSC on a unit base by assessing DC.

### What this adds to what was known

Our finding in Task 1 (Cronbach’s α was insufficient to a scale’s validity) was consistent with the literature [40, 41]: Internal consistency is a necessary but not sufficient condition for measuring homogeneity or unidimensionality in a sample of test items. Thus, an extremely low Cronbach’s α refers to a poor validity (i.e., necessary condition). By contrast, a high Cronbach’s α is not always related to a good validity (i.e., sufficient condition; see Fig. 3). Given the limitations of Cronbach’s α, a different but more conservative measure of internal consistency reliability (i.e., the composite reliability) should be applied because it considers the different outer loadings of the indicator variables [42].

Reports [43–45] about the acceptable values of Cronbach’s α (from 0.70 to 0.95) are inconsistent. The number of item length, item interrelatedness, and dimensionality affect the value of alpha [46]. We simulated scenarios with item length from 5 to 100 and found that (i) a low alpha could be due to a low number of items (=5), poor interrelatedness (=0.3) between items, or heterogeneous constructs [47] (Fig. 3, left); and (ii) a high alpha (>0.9) implies that some items were redundant as they were testing the same question (=100) but in a different guise [47]. A maximum alpha of 0.90 has been recommended [48].

### What it implies and what should be changed?

Our finding in Task 2 (DC combined with PA to inspect the number of factors for a scale) is congruent with a previous study [17] that suggested incorporating Cronbach’s α with DC to jointly assess a scale’s quality. We also responded to the argument [49] that using Cronbach’s α often is related to the PCA approach in practical test construction, especially when factor loadings are not easily obtained in MS Excel.

Referring to the literature [50], composite reliability (CR)=$\frac{{\left(\sum {\lambda}_i\right)}^2}{{\left(\sum {\lambda}_i\right)}^2+\left(\sum {\varepsilon}_i\right)}$, where λ (lambda) is the standardized factor loading for item i, and ε is the respective error variance for item i. However, it is hard to gain the CR for a scale using MS Excel. We suggest that DC reached an equivalent effect in comparison with CR (≥0.70) as a criterion to measure rigorous internal consistency reliability [42]. DC combined with PA to examine a scale’s unidimensionality significantly improved classification precision (Fig. 5).

### Strengths of this study

In Task 3, we applied an author-made MS Excel module to examine the validity of a PSC domain in a unit base. Such a tool is rarely reported in previous papers. We also demonstrated five videos in Additional files for interested readers to easily understand (i) how to extract PA 95% CI eigenvalues from the internet, (ii) how to manipulate scenarios under requirements of the Rasch model and generate a dual dimension interrelation for true scores based on the literature [51] using a rotation of axes with a trigonometric function: true score * cos(RADIANS(angle) + random dataset* sin(RADIANS(angle), (3) how to quickly complete the computation for 100 hospital units at a single time to examine the validity of PSC domains using the DC detection technique, and (4) how to draw ROC curves with MedCal statistical package (see Additional file 5). In addition, the MS Excel module we programmed could enhance the article, through which future researchers can imitate our methodology to simulate their own data and verify their own results.

### Limitations and future study

Our study had some limitations. First, only two PA methods of 95% PA random data and individual eigenvalues were used to compare the accuracy. Results showed that PA was merely accurate at a medium item length and sample size (see Additional file 3). Readers are recommended to replace the 95% PA random data eigenvalues in the spreadsheet [eigen] in Additional file 6 with other alternatives for further investigations using the extraction technique shown in Additional file 1 in the future.

Second, we compared the observed eigenvalues with those obtained from uncorrelated normal variables. The result might be different if random eigenvalues were obtained from data of known factorial structure [52]. The finding that PA was merely accurate at a medium item length and sample size from this study could not refute the argument from various studies [21, 53]. PA is the most accurate, showing the least variability and sensitivity to different factors. However, such results should be further verified in the future.

Third, we demonstrated PSC validity in a hospital unit and found that the global subscales with low construct validity (<0.70, first row in Table 1) were teamwork climate (DC = 0.58) and working conditions (DC = 0.66). Those misfit items emerging in data could not be used to make an inference on the issue of data purification or data entry errors with seriously cheating and careless behaviors, ora DIF [39] phenomenon possibly occurring among hospitals, which were not investigatged in the previous published paper [6]. .

## Conclusion

PA is not well known among researchers partly because it is not included as an analysis option in most professional statistical packages. This study provides an alternative user-friendly application (a Microsoft Excel tool) that can determine whether a scale is one-dimensional once DC has been applied to examine scale unidimensioality. Such findings support the idea of combining PA and DC to jointly assess a scale’s quality in healthcare settings.

## Additional files

Additional file 1: MS Excel module extracting data from website. http://www.healthup.org.tw/marketing/course/marketing/extrating_eigen.mp4. (MP4 5908 kb)

Additional file 2: MS Excel module simulating data under requirements of the Rasch model. http://www.healthup.org.tw/marketing/course/marketing/PSC_rasch_simulate.mp4 (MP4 13678 kb)

Additional file 3: Comparisons of resulting patterns across scenarios from the three methods. (XLSX 43 kb)

Additional file 4: MS Excel module for calculating unit DC and Cronbach’s α on PSC data for a hospital. http://www.healthup.org.tw/marketing/course/marketing/PSC_data_demo.mp4 (MP4 7223 kb)

Additional file 5: Draw a ROC curve using MedCal. http://www.healthup.org.tw/marketing/course/marketing/ROC_MedCal.mp4 (MP4 4972 kb)

Additional file 6: Resulting data and the author-made module. (XLSM 1782 kb)

## Abbreviations

AUC: Area under receiver operating characteristic curve
CI: Confidence interval
CR: Composite reliability
DC: Dimension coefficient
EFA: Exploratory factor analysis
PA: Parallel analysis
PCA: Pricipal component analysis
PSC: Patient safety culture survey
VBA: Visual Basic for Applications

## References

[1] Willmott J, Mould J. Health professionals.Aust Health Rev. 2017 30. doi:10.1071/AH16274.

[2] Kohn LT, Corrigan JM, Donaldson MS (2000). To err is human: building a safer health system.

[3] Leape LL (1994). Error in medicine. JAMA.

[4] Vincent C, Taylor-Adams S, Stanhope N (1998). Framework for analyzing risk and safety in clinical medicine. BMJ.

[5] Reason J (1995). Understanding adverse events: human factors. Qual Health Care.

[6] Lee WC, Wung HY, Liao HH, Lo CM, Chang FL, Wang PC, Fan A, Chen HH, Yang HC, Hou SM (2010). Hospital safety culture in Taiwan: a nationwide survey using Chinese version safety attitude questionnaire. BMC Health Serv Res.

[7] Chien TW, Shao Y, Kuo SC (2017). Development of a Microsoft excel tool for one-parameter Rasch model of continuous items: an application to a safety attitude survey. BMC Med Res Methodol.

[8] Pimentel MPT, Choi S, Fiumara K, Kachalia A, Urman RD. Safety Culture in the Operating Room: Variability Among Perioperative Healthcare Workers. J Patient Saf. 2017. doi:10.1097/PTS.0000000000000385. 10.1097/PTS.0000000000000385 28574955

[9] Colla JB, Bracken AC, Kinney LM, Weeks WB (2005). Measuring patient safety climate: a review of surveys. Qual Saf Health Care.

[10] Elsous A, Akbarisari A, Rashidian A, Aljeesh Y, Radwan M, Abu Zaydeh H. Psychometric properties of an Arabic safety attitude questionnaire (short form 2006).Oman Med J 2017;32(2):115-123. doi:10.5001/omj.2017.21. 10.5001/omj.2017.21 PMC5397085 28439381

[11] Rudner L, Wright BD (1995). Diagnosing person misfit. Rasch Meas Trans.

[12] Çokluk Ö, Koçak D. Using Horn’s parallel analysis method in exploratory factor analysis for determining the number of factors. Educational Sciences: Theory & Practice. 2016;16:537–51.

[13] Kline RB. Principles and practice of structural equation modeling. New York & London: The Guilford Press; 2005.

[14] Nunnally JC, Bernstein IH (1994). Psychometric theory.

[15] Fabrigar LR, Wegener DT, MacCallum RC, Strahan EJ (1999). Evaluating the use of exploratory factor analysis in psychological research. Psychol Methods.

[16] Hayton JC, Allen DG, Scarpello V (2004). Factor retention decisions in exploratory factor analysis: a tutorial on parallel analysis. Organ Res Methods.

[17] Henson RK, Roberts JK (2006). Use of exploratory factor analysis in published research: common errors and some comment on improved practice. Educ Psychol Meas.

[18] Horn JL (1965). A rationale and test for the number of factors in factor analysis. Psychometrica.

[19] Silverstein AB (1977). Comparison of two criteria for determining the number of factors. Psychol Rep.

[20] Silverstein AB (1978). Note on the parallel analysis criterion for determining the number of common factor or principal components. Psychol Rep.

[21] Zwick WR, Velicer WF (1986). Comparison of five rules for determining the number of components to retain. Psychol Bull.

[22] Ledesma DR, Mora PV (2007). Determining the number of factors to retain in EFA: an easy-to-use computer program for carrying out parallel analysis. Practica Assess Res Eval.

[23] Crawford AV, Green BS, Levy R, Lo WJ, Scott L, Svetina D, Thompson M (2010). Evaluation of parallel analysis methods for determining the number of factors. Educ Psychol Meas.

[24] Patil VH, Surendra NS, Sanjay M, Todd D (2008). Efficient theory development and factor retention criteria: a case for abandoning the ‘Eigenvalue greater than one’ criterion. J Bus Res.

[25] Patil VH., Surendra NS, Sanjay M, Todd D. Parallel Analysis Engine to Aid Determining Number of Factors to Retain [Computer software]. Available from http://smishra.faculty.ku.edu/parallelengine.htm

[26] O'Connor BP (2000). SPSS and SAS programs for determining the number of components using parallel analysis and Velicer's MAP test. Behav Res Methods Instrum Comput.

[27] Chien TW (2012). Cronbach's alpha with the dimension coefficient to jointly assess a scale's quality. Rasch Meas Trans.

[28] Rasch G (1960). Probabilistic models for some intelligence and attainment tests.

[29] Downing SM (2003). Validity: on the meaningful interpretation of assessment data. Med Educ.

[30] Feldt LS, Brennan RL, Linn RL (1989). Reliability. Educational measurement.

[31] Andrich D (1978). A rating formulation for ordered response categories. Psychometrika.

[32] Linacre JM (2007). How to simulate Rasch data. Rasch Meas Trans.

[33] Lord FM (1980). Applications of item response theory to practical testing problems.

[34] Divgi DR. Dimensionality of binary items: Use of a mixed model. Paper presented at the annual meeting of the National Council on Measurement in Education. Boston, MA, 1980.

[35] Young FW. ViSta : the Visual Statistics System”. [computer software] [on-line] 2017/6/4 available at http://forrest.psych.unc.edu/research/index.html

[36] Tennant A, Pallant JF (2006). Unidimensionality matters! (a tale of two Smiths?). Rasch measurement. Transactions.

[37] Chien TW, Chang Y, Chien PS, Lin HJ (2015). A dashboard used for displaying the results of the hospital patient safety culture survey. J Taiwan Assoc Med Inform.

[38] Eisinga R, Te Grotenhuis M, Pelzer B (2013). The reliability of a two-item scale: Pearson, Cronbach or spearman-Brown?. Int J Public Health.

[39] Holland PW, Wainer H (1993). Differential item functioning.

[40] Cortina J (1993). What is coefficient alpha: an examination of theory and applications. J Appl Psychol.

[41] Green S, Lissitz R, Mulaik S (1977). Limitations of coefficient alpha as an index of test unidimensionlity. Educational psychological. Measurement.

[42] Hair Jr JF, Hult GTM, Ringle CM, Sarstedt M. A Primer on Partial Least Squares Structural Equation Modeling (PLS-SEM). Thousand Oaks, California : Sage, 2017. Publications.

[43] Nunnally J, Bernstein L (1994). Psychometric theory.

[44] Bland J, Altman D (1997). Statistics notes: Cronbach's alpha. BMJ.

[45] DeVellis R (2003). Scale development: theory and applications: theory and application.

[46] Panayides P. Coefficient Alpha. Europe's Journal of Psychology. 2013;9(4):687–96.

[47] Tavakol M, Dennick R. Making sense of Cronbach’s alpha. Int J Med Educ. 2011;2:53–5. 10.5116/ijme.4dfb.8dfd PMC4205511 28029643

[48] Streiner D (2003). Starting at the beginning: an introduction to coefficient alpha and internal consistency. J Pers Assess.

[49] Sijtsma K (2009). On the use, the misuse, and the very limited usefulness of Cronbach's alpha. Psychometrika.

[50] Jöreskog KG, Sörbom D (1996). LISREL 8 User's reference guide.

[51] Smith RM, Miao CY. Assessing unidimensionality for Rasch measurement. Chapter 18 in M. Wilson (Ed.) Objective Measurement: Theory into Practice 1994; 2, Norwood NJ: Ablex.

[52] Ruscio J, Roche B (2012). Determining the number of factors to retain in an exploratory factor analysis using comparison data of known factorial structure. Psychol Assess.

[53] Humphreys LG, Montanelli RG (1975). An investigation of the parallel analysis criterion for determining the number of common factors. Multivar Behav Res.

